# The coin model of privilege and critical allyship: implications for health

**DOI:** 10.1186/s12889-019-7884-9

**Published:** 2019-12-05

**Authors:** Stephanie A. Nixon

**Affiliations:** 1 Department of Physical Therapy, University of Toronto, 160-500 University Avenue, Toronto, ON M5G 1V7 Canada; 2 Dalla Lana School of Public Health, University of Toronto, 155 College St Room 500, Toronto, ON M5T 3M7 Canada

**Keywords:** Health equity, Social determinants of health, Social justice, Privilege, Oppression, Intersectionality, Racism, Indigenous health, Ableism, Allyship

## Abstract

Health inequities are widespread and persistent, and the root causes are social, political and economic as opposed to exclusively behavioural or genetic. A barrier to transformative change is the tendency to frame these inequities as unfair consequences of social structures that result in disadvantage, without also considering how these same structures give unearned advantage, or privilege, to others. Eclipsing privilege in discussions of health equity is a crucial shortcoming, because how one frames the problem sets the range of possible solutions that will follow. If inequity is framed exclusively as a problem facing people who are disadvantaged, then responses will only ever target the needs of these groups without redressing the social structures causing disadvantages. Furthermore, responses will ignore the complicity of the corollary groups who receive unearned and unfair advantage from these same structures. In other words, we are missing the bigger picture. In this conceptualization of health inequity, we have limited the potential for disruptive action to end these enduring patterns.

The goal of this article is to advance understanding and action on health inequities and the social determinants of health by introducing a framework for transformative change: the Coin Model of Privilege and Critical Allyship. First, I introduce the model, which explains how social structures produce both unearned advantage and disadvantage. The model embraces an intersectional approach to understand how systems of inequality, such as sexism, racism and ableism, interact with each other to produce complex patterns of privilege and oppression. Second, I describe principles for *practicing critical allyship* to guide the actions of people in positions of privilege for resisting the unjust structures that produce health inequities. The article is a call to action for all working in health to (1) recognize their positions of privilege, and (2) use this understanding to reorient their approach from saving unfortunate people to working in solidarity and collective action on systems of inequality.

## Background

The narrator at the start of a YouTube video instructs, “Count how many times the team wearing white passes the ball” [1]. Six people enter the screen: three wearing white shirts, and three wearing black. They stand in a circle and pass basketballs to each other for 90 seconds. At the end of the video, the narrator says, “The correct answer is 16 passes. Did you spot the gorilla?” A portion of the audience typically misses the gorilla and is baffled by the question [2]. The video then repeats to show again that in the midst of the basketball passing, an adult dressed as a gorilla walks into scene, looks at the camera thumping his chest, then leaves. This short exercise demonstrates how it is possible to miss something as obvious as a gorilla, but also invites the audience to imagine how it could be otherwise. That is, the audience had their capacity built to count the number of passes by the team wearing white whereas the narrator could have built the capacity of the audience to spot the gorilla. So it is with privilege: that it is possible to miss something as obvious as an adult in a gorilla suit walking into the screen. It follows that if one missed the gorilla (or privilege), then there is no possibility of engaging in conversation about the gorilla. One might even question the legitimacy of other people’s claims about the existence of a gorilla (or privilege). However, the narrator could have built the capacity of the audience to see the gorilla, and so too can people build their capacity to see privilege [3]. Building this capacity requires both learning and unlearning, and is the aim of this article.

This article provides a framework, the Coin Model of Privilege and Critical Allyship, for conceptualizing privilege in order to address the even more important question of what to do about it, which is termed *practicing critical allyship*. In particular, I explore how privilege and allyship are related to health inequity – that is, disparities that are systemic, avoidable, and unfair [4]. Efforts to address inequities tend to frame concerns as unfair consequences of social structures that result in poor health, without consideration of the ways in which these same social structures give unearned advantage to others. Unearned advantage, or privilege, is the gorilla. Practicing critical allyship is the orientation to guide action for people who find themselves in positions of privilege in relation to a particular system of inequality.

Disappearing privilege from discussions of health equity is an important shortcoming, because the framing of a problem sets the universe of possible solutions that will follow. If inequity is framed exclusively as a problem facing people who are marginalized, then responses will only attempt to address the needs of these groups, without redressing the social structures causing this disadvantage, or the complicity of the corollary groups who receive unearned (and unfair) advantage from these same structures [5 ,6]. This approach, often called anti-oppression [7], is well developed in other fields [8–11] but less so within health research [12]. To be clear, the ideas presented in this article about privilege and oppression are not new; they have been articulated, advanced and argued for decades, largely by “marginalized” groups to people in positions of privilege who have not been ready or willing to listen. In this article, I seek to translate these ideas in a new way for a general health audience, and it is noteworthy that successful uptake of the ideas in this version must be understood as inextricably linked to my position as someone “on the top of many coins”.

The first part of this article introduces the metaphor of the coin, a framework for understanding how social structures offer both unearned advantage (‘privilege’) and disadvantage (‘oppression’). I adapted the coin metaphor from the schematic of privilege, domination and oppression presented by Kathryn Pauly Morgan in “Describing the Emperor’s New Clothes: Three Myths of Educational (In)Equality” [13]. The Coin Model of Privilege and Critical Allyship aims to inform action to resist and dismantle the unjust structures that produce inequities. The model embraces an intersectional approach to consider how systems of inequality, such as racism, heterosexism and ableism, interact to produce complex patterns of unearned disadvantage and advantage. The second part of this article introduces principles for *practicing critical allyship* and their implications for mitigating health inequities.

## The coin model

### The coin

There are norms, patterns and structures in society that work for or against certain groups of people, which are unrelated to their individual merit or behaviour. Put another way, there are (often invisible) systemic forces at play that privilege some social groups over others, such as sexism, heterosexism, racism, ableism, settler colonialism, and classism [14]. These unfair social structures have profound effects on health, producing inequities in morbidity and mortality.

Racism is well demonstrated to adversely affect the health of non-white people through interconnected structural, institutional, cultural and psychosocial pathways [15, 16]. For instance, there is a breadth of evidence in the American context demonstrating that people who are racialized receive lower quality health services and are less likely to receive routine medical procedures than white Americans [17]. Racism and its interconnection with colonialism have created profound health inequities for Indigenous Peoples, including lower life expectancy (by more than 5 years) than the non-Indigenous population in the United States [18–20]. Women and girls have worsened health outcomes, diminished capacity to realize health-related human rights, and reduced access to healthcare, which are related to sexism, and its intersections with class, race and ability [21–23]. People who are gay, lesbian or bisexual face health inequities related to heteronormativity and homophobia [24, 25]. Furthermore, there is worsened health among transgender people due to cisnormativity and transphobia, which is exacerbated by other systems of oppression [23, 26–27]. A study in the Canadian province of Ontario found that one in ten trans people who had accessed an emergency room had been refused care or had care terminated prematurely because they were trans, and 40% had experienced discriminatory behaviour from a family doctor [27]. A final example is health disparities among people with disabilities related to ableism and its intersections with other systems of inequality [28, 29]. Census data from 2015 demonstrated that nearly 14% of Australians with a disability reported disability-based discrimination in the previous year; that disability-based discrimination was more common among people who were unemployed or poor; and, that disability-based discrimination was associated with higher levels of psychological distress and poorer self-reported health [29]. These systems of inequality are bad for health.

In the Coin Model, each system of inequality is conceptualized as a coin. Coins do not reflect the individual behaviour of good or bad people. Rather, they are society-level norms or structures that give advantage or disadvantage regardless of whether individuals want it or are even aware of it. Each coin represents a different system of inequality.

These social structures, or coins, give unearned advantage or disadvantage according to one’s relationship to that particular system of inequality. For instance, one may consider the coin (or system of inequality) of heterosexism. Heterosexuality is romantic or sexual attraction to people of the opposite sex. Heterosexism, a dominant norm in many societies, views being heterosexual as the only normal and right way to be. People who happen to fit this norm because they are straight (i.e., heterosexual) enjoy advantages from this social structure. For instance, they can openly express affection without fear of discrimination or violence. They see their way of life validated and valued through its regular, positive, and default position as the normal way of being reflected in legal frameworks and popular culture. However, straight people did not choose to be straight; they just are. They did not earn this advantage; rather, they lucked into it by their natural preference for whom they love being in alignment with this broader social norm. They likely did not ask for these benefits, but they receive them all the same. They may not even be aware that they are receiving unearned advantage, but they receive it nonetheless [30].

Conversely, people who are not straight do not enjoy this freedom from discrimination and violence, or the sense of inclusion and belonging that results from this social structure. People who are not straight, such as people who identify as gay, lesbian, bisexual, asexual, or two-spirit, did not choose to be that way; they just are. However, their natural preference for whom they love is *not* in alignment with the dominant norm of heterosexism and, as such, they receive unearned disadvantage. They did nothing to earn it, but they receive it nonetheless. Furthermore, while unearned advantage can be difficult to see, unearned disadvantage is often highly visible to those who experience it.

### The bottom and top of the coin: oppression and privilege

**Fig. 1:**
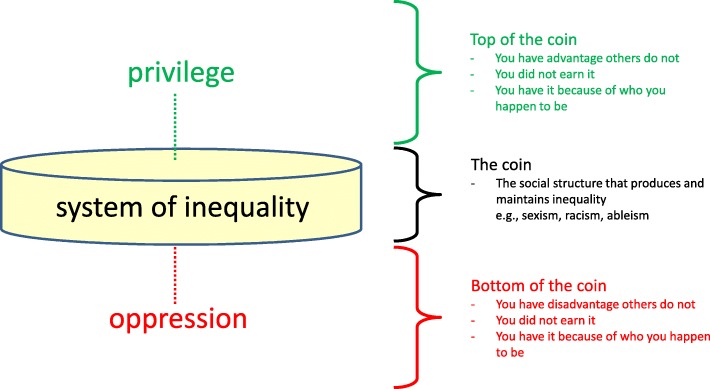
The coin

It is the same social structure, or coin, that gives unearned disadvantage to some and unearned advantage to others. Groups of people who are disadvantaged by this social structure are viewed as being on *the bottom of the coin* (see Fig. 1). In this model, I call this side of the coin *oppression*. Because of the dire health effects resulting from this unfair disadvantage, these are the groups commonly targeted in health promotion research and interventions. The names for these groups are many and familiar, including marginalized populations, disadvantaged groups, vulnerable communities, high-risk groups, priority neighbourhoods, or hard-to-reach populations.

Other groups of people receive advantage from these same social structures, and are viewed as being on the *top of the coin*. These groups receive benefits from the structures that others do not, which they did not earn. Rather, they receive the benefit because they luck into being in alignment with the norms of that particular social structure. In this model, I call the position on the top of the coin *privilege*.

Terms used to describe groups of people who enjoy unearned health benefits as a result of systems of inequality are uncommon and hard to imagine (e.g., unfairly advantaged groups, free-lift populations). To view those on the top of the coin as “normal” or “average patients” is erroneous since, by definition, the top of the coin represents people who are the recipients of unearned and unfair benefits because their way of being is valued over others. The goal is not to move people from the bottom of the coin to the top, because both positions are unfair. Rather, the goal is to dismantle the systems (i.e., coins) causing these inequities.

Drawing attention to the top of the coin is important because inequity is relational: the bottom of the coin is disadvantaged compared to the top. Yet, issues of health equity are often framed exclusively as problems facing people on the bottom of the coin. Disappearing the top of the coin, and often the coin itself, functions to maintain the status quo because what one frames as the problem sets the universe of conceivable actions to address it. When the problem is framed as challenges faced by the members of a “vulnerable group” (i.e., bottom of the coin), then potential solutions will focus exclusively on interventions to address their issues. Should actions address the needs of these groups? Of course; these responses are deeply important for redressing existing inequities. However, the bottom of the coin is commonly framed as the entire story of health equity as opposed to just one part. If the problem was viewed not only as the bottom of the coin, but also the coin itself (i.e., the unjust social structure that gives unearned disadvantage to people on the bottom), then a different set of solutions could follow, such as changes to policy and law to create safeguards against discrimination produced by the system of inequality. Indigenous physician and public health leader, Marcia J. Anderson, succinctly captures this point as follows:

“From now on instead of ‘vulnerable people’ I'm going to use the phrase ‘people we oppress through policy choices and discourses of racial inferiority.’ It's a bit longer but I think will help us focus on where the problems actually lie.” [31]

For instance, the coin of ableism reflects the social structure that discriminates against disabled people in favour of people who fit a socially-constructed norm of able-bodiedness [32]. In an ableist worldview, there is a particular version of ability that is assumed to be normal or natural (top of the coin), and people who cannot meet this expectation (bottom of the coin) are viewed as a problem who should strive to become, or assimilate to, the norm. Ableism views disability as a mistake or failing rather than a simple consequence of human diversity, like sexual orientation or gender.

Consider the different solutions that become imaginable depending on whether one views the problem as the bottom of the coin (i.e., disabled people) or the coin itself (i.e., ableism). Solutions addressing the bottom of the coin strive to support disabled people to achieve the norm of able-bodied people, including medical care and rehabilitation to fix disability within the body. Conversely, if one views the problem as the unfair social structure of ableism, then the cause of disability shifts: instead of being located within an individual’s body, disability is understood as resulting from the social, attitudinal and political environment. Responses become focused on social change to achieve equity for people with disabilities in the same light as equity for other disadvantaged groups where prejudice, segregation, and inaccessibility are viewed as the problem. Responses might focus on rights-based approaches aligned with the United Nations Convention on the Rights of Persons with Disabilities. Actions would shift from focusing on disability as a mistake to instead celebrating difference by creating flexible systems (e.g., through policies, the built environment) that enable and liberate as opposed to disable and exclude.

Problematizing the coin of ableism also shines a spotlight on the profoundly disabling effects of stigmatizing attitudes commonly held by able-bodied people. In many cases, such effects are unintended and unknown to those reproducing them, but profoundly impactful all the same, which brings us to the top of the coin.

### Seeing the gorilla: recognizing the effects of invisiblizing privilege

The coin of settler colonialism in the context of Canada provides another useful illustration. If the coin is settler colonialism, then the group receiving unearned disadvantage on the bottom of that coin is Indigenous Peoples. Since the Idle No More movement and 2015 report of the Truth and Reconciliation Commission of Canada, the history and legacy of colonization are starting to be recognized within Canadian society [33, 34]. For instance, there is greater attention to the ongoing, devastating effects of Indian Residential Schools on Indigenous Peoples, the harmful effects of the Government of Canada’s Indian Act, and the rights-violations embedded in the inequitable provision of public funding to ensure basic determinants of health (e.g., clean drinking water, quality primary education) within Indigenous communities. These examples draw attention to the coin (i.e., settler colonialism) as the source of profound health inequities between Indigenous and non-Indigenous people in Canada. The problem has been relocated from Indigenous People (the bottom of this coin) to the structures (the coin) that create the conditions that produce unearned and unfair disadvantages. The growing ability to see, and thereby devise solutions to address, the coin is an important marker of progress toward dismantling this inequity.

But Indigenous People and settler colonialism are not the complete picture. Similarly, disabled people (bottom of the coin) and ableism (the coin), are not the complete picture. What about the people on the tops of these coins? Who are they? What is their role in dismantling, or as is often the case, unintentionally strengthening the coin?

A key task for people who find themselves on the top of a coin is to see the gorilla; that is, to understand that there is a coin, that it has two sides, and that they occupy the position of unearned advantage (i.e., privilege) on the top. For instance, if Indigenous People are on the bottom of the coin, it is non-Indigenous people (often referred to as settlers) who receive unearned and unfair advantage from these same structures. Seeing the gorilla in this instance means developing the capacity to ask and answer questions such as, “In which ways did I benefit from settler privilege today?” and “In what ways did my actions today reflect and thereby reinforce the coin of settler colonialism?”

In many cases, people on the top of a coin did not ask for the unearned advantage that they receive. However, people are rarely on the top of the coin because of merit or worth (commonly referred to as the myth of meritocracy [35]). Rather, they are there, by definition, because they happen to be able-bodied, settlers, white, straight, cisgender, or other aspects of their social identity that they did not choose, but which nonetheless align with historic planes of domination and subordination [13].

Just as the disadvantage received by people on the bottom of the coin is unearned and unfair, so too, the advantage received by people on the top of the coin is unearned and unfair. However, these opposite effects of the coin are not evenly understood.

### The contradiction of who holds expertise vs who holds power regarding systems of inequality

The unfair disadvantage associated with the bottom of the coin is frequently in plain view – to clinicians and researchers working to address these challenges, and especially to people on the bottom of the coin themselves who may confront these disadvantages daily. Regardless of whether people on the bottom of the coin are fluent in the language of anti-oppression, they typically are expert in the many ways that the coin operates to create disadvantage, dehumanization, lack of safety and social exclusion. Moreover, it is these groups who have historically led movements to dismantle the coins, such as Indigenous Peoples leading movements to redress the harmful effects of colonization on First Peoples and the environment, or Black people leading anti-racism civil rights movements.

However, the unearned advantage associated with being on the top of the coin is often invisible – in health promotion interventions, in health equity research, and especially to the people themselves who occupy positions on the top of coins. Some have argued that the obliviousness of people about their positions of privilege is a key strategy required to sustain the hegemony of systems of inequality [36]. Learning to see the gorilla is a strategy for becoming less oblivious and less harmful.

Lack of awareness about the top of the coin has serious implications for meaningfully addressing health equity. This is because lack of recognition of the societal influences that have helped elevate people on the top of coins to reach their professional, economic or social positions commonly leads those same people to presume that they are there exclusively because of their individual merit. Put another way, where privilege is unchecked, it can lead to an irrational sense of entitlement, expertise and access. It then seems logical and, indeed, a moral imperative for those on the top of the coin to be guided by an altruistic urge to save or fix people on the bottom of the coin. However, this logic no longer holds when one considers who possesses expertise regarding the coin and its effects; that is, people on the bottom of coins.

Furthermore, invisibilizing the top of the coin allows people in positions of privilege to view themselves as unconnected to, or outside of, the systems of inequality they are trying to address, as opposed to understanding their direct relationship to people on the bottom of the coin. Instead of understanding their complicity within systems of inequality, disappearing the top of the coin allows people on the top to frame their role in health equity work as neutral, selfless and altruistic. This positioning logically leads to action that (exclusively) assists people on the bottom of the coin as opposed to targeting oppressive systems that are bad for all.

Within the health sphere, the people who typically hold the power to allocate resources, design programs, and draft policy to address the needs of people on the bottom of the coin often find themselves on the top of multiple coins. But who are the real experts in understanding how the coin operates in society? When people in privilege do not realize the powerful implications of that position, they may unwittingly – and with the best of intentions – devote themselves to trying to help people on the bottom without ever understanding: (1) the impact of the coin on their own individual position, (2) how this lack of understanding vastly compromises their insight about the oppressive social structure, and (3) how this lack of insight can lead to actions that serve not to dismantle the coin, but to strengthen the status quo. For instance, the assumed expertise of people on the top of the coin to solve the problems of inequity becomes reinforced, while the assumed neediness and lack of expertise of people on the bottom of coins is further entrenched. Materials resources (e.g., salaries, grant funding) to address health equity commonly flow to people on the top of the coin to design and administer programs for people on the bottom of the coin, thus reinforcing inequities.

In summary, lack of awareness about one’s position on the top of coins is dangerous for health equity. Indeed, the invisibility of privilege is central to the functioning and sustainability of the system of inequality. Invisiblilizing the top of the coin, and frequently the coin itself, ensures that the coin remains strong. This is the gorilla, and why movement toward dismantling systems of inequality requires everyone, and especially people on the top of coins, to learn how to see the gorilla.

### Recognizing the intersecting nature of multiple coins

A single coin does not represent all privilege or all oppression. Rather, each coin represents a specific system of inequality (e.g., sexism, racism, ableism). Each person typically occupies the position on the top of some coins and the bottom of other coins at the same time. A common pattern is for people to have a well-developed understanding of the system of inequality for which they find themselves on the bottom and, perhaps, frustration, anger or sadness that this unjust system is not better understood by people on the top of that same coin. This insight can be helpful for then considering one’s (often limited) knowledge about the systems of inequality where they find themselves on the top.

**Fig. 2:**
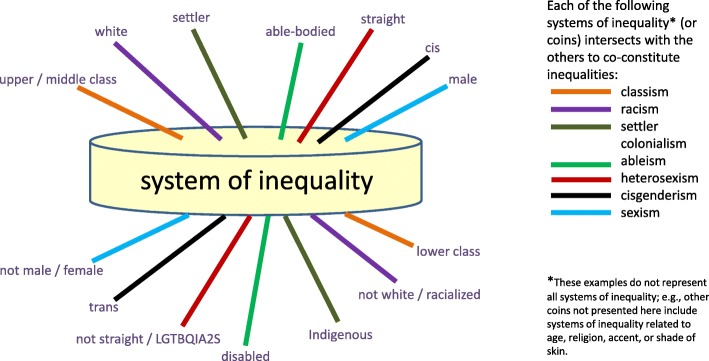
The intersecting nature of the coins, which produces complex patterns of advantage and disadvantage

Furthermore, it is important to recognize that while each coin represents a different system of inequality, the coins do not operate in isolation. Rather, the coins *intersect* to create complex inter-relating systems of inequality (see Fig. 2). The result is not additive; finding oneself on the same side of two coins does not mean that one is twice as privileged or twice as oppressed. Rather, intersecting systems of inequality produce new and complex patterns of advantage and disadvantage. The relevance and impact of these positions varies according to context, and so one’s positions on these multiple coins need to be analyzed together. The term, *intersectionality*, was introduced by legal scholar and critical race theorist, Kimberlé Crenshaw, and further understood as the matrix of domination by Black feminist scholar, Patricia Hill Collins, in order to characterize the unique forms of oppression faced by women who are Black [37, 38]. Intersectionality has been taken up widely, including within the health sphere [39, 40].

Analysis requires the precision of clarifying one’s position on the top or bottom of each particular coin, with special attention to those coins for which one is on top, and how these individual positions may amplify each other in different contexts. Importantly, not all coins are the same size; that is, different systems of inequality will matter more or less in different contexts, and depending on their intersection with other patterns of inequality.

Another key insight offered by an intersectional analysis is how experiences of oppression in one system of inequality do not negate positions of privilege in others. For instance, a white person who is poor may clearly understand the oppressive effects of classism, but may not also appreciate the ways they simultaneously benefit from being on the top of the coin of racism. A racialized person who is considered able-bodied may understand the devastating effects of racism while being unaware of how their ableist privilege serves to regularly give them unearned advantage. An intersectional analysis reminds us that the effects of these different positions cannot be understood through a mathematical approach whereby the position on the bottom of one coin cancels out the position on the top of another. This is how even the most articulate activists on certain systems of inequality can unintentionally strengthen other coins where they find themselves on top because of their unrecognized positions of privilege, i.e., their lack of capacity to see that particular gorilla.

### This is not about innocence or guilt

Discussions of privilege can lead to faulty assumptions of innocence, and counterproductive attention to guilt. The coin model is premised on an analysis that rejects both of these unhelpful patterns.

Framing people on the top of the coin as oblivious about their unearned privilege does not equate to innocence among those individuals. For the most part, people within the health sphere who are in positions of privilege do not intend to cause harm; however, these coins were created very intentionally by people on the top of the coin. These systems were designed to oppress, and they are sustained, intentionally by some and unintentionally by others, who are on top of the coin. It is not the *intent* of one’s actions that matters but the *impact*, and the impact of oblivion among people on the top of the coin can be deeply harmful, dehumanizing and violent to people on the bottom of the coin. Indeed, these systems of inequality are harmful to whole societies because they diminish the contributions and talents of people on the bottom of coins through the barriers they face.

Another common narrative is the feeling of guilt among people when considering the unearned benefits they receive because of being on the top of a coin. Feelings of guilt can lead to discomfort, distancing from the issue, denial, or intellectual paralysis. In the context of racism, white academic Robin DiAngelo calls this phenomenon “white fragility” [41]. Guilt can become the primary focus of discussion and analysis among people who share positions on the top of a coin. However, the coin model invites analysis of how focusing on guilt serves to *strengthen* vs dismantle systems of inequality. Guilt leads to feelings of distress among people through reflecting on the unearned advantages and free lifts that make their lives easier. This distress must be understood in contrast to the (often daily) distress, dehumanization, and violence experienced by people on the bottom of the coin. Furthermore, focusing on the guilt born of discovering unearned benefits serves to centre the needs and feelings of people on the top of the coin, which reinforces the coin by crowding out the needs and feelings of people on bottom. In the words of the Black, lesbian poet and philosopher, Audre Lorde:

“Guilt is not a response to anger; it is a response to one’s own actions or lack of action. If it leads to change then it can be useful, since it is then no longer guilt but the beginning of knowledge. Yet all too often, guilt is just another name for impotence, for defensiveness destructive of communication; it becomes a device to protect ignorance and the continuation of things the way they are, the ultimate protection for changelessness.”[42]

If guilt is an unproductive strategy for people on the top of the coin who wish to dismantle inequities, then what might be alternatives? A more productive strategy is to recognize feelings of guilt, and swiftly reframe guilt as responsibility deriving from complicity [43]. Embracing responsibility gives rise to action to resist the dominant norms that sustain systems of inequality, which I refer to as practicing critical allyship.

## Principles for practicing critical allyship

To work toward dismantling a system of inequality, different orientations are appropriate depending on one’s position in relation to that social structure; that is, the side one is on of a particular coin. This article is written for people who find themselves on the top of a coin that they wish to dismantle (i.e., in a position of privilege), and I call this orientation *practicing critical allyship*.

Within this approach, allyship is not an identity, but an ongoing practice. The focus on critical allyship as a *practice* aligns with the focus by others on *becoming* an ally [44–46], or what queer Black author Mia McKenzie describes as “currently acting in solidarity with” to focus on actions in the present [47]. This approach learns from the Anti-Oppression Network, which defines allyship as an “active, consistent, and arduous practice of unlearning and re-evaluating in which a person of privilege seeks to operate in solidarity with a marginalized group of people” [48].

Practicing critical allyship requires a reorientation from the dominant way of thinking about how people in positions of privilege should address inequities, which assumes that the most ethical and effective way to address health disparities is for people on the top of the coin to use their expertise to help marginalized groups with their problems. “Their problems” are typically framed as caused by the behaviours of individuals or groups, as opposed to the being linked to unearned disadvantage resulting from systems of inequality (i.e., the coin). It follows that appropriate responses involve people on the top of the coin going into communities (locally and overseas) to bring their expertise and solutions to needy individuals. Although often well-intentioned, people on the top of the coin have been socialized to have little understanding of their relationship to social structures, or the contradiction between who holds power versus who holds expertise. This flawed approach strengthens the system of inequality by reinforcing the assumption that people on the top are experts, unconnected to the system of inequality, and that people on the bottom need rescuing. If the goal is to dismantle the systems of inequality that result in health disparities, a reorientation is required whereby people on the top of the coin reorient their motivation from:

-
  - I wish to help the less fortunate, or
  - I use my expertise to reduce inequities for marginalized populations
- to the following commitments:
  - I seek to understand my own role in upholding systems of oppression that create health inequities.
  - I learn from the expertise of, and work in solidarity with, historically marginalized groups to help me understand and take action on systems of inequality.
  - This includes working to build insight among others in positions of privilege, and *mobilizing in collective action* under the leadership of people on the bottom on the coin.

This reframing reverses who is presumed to be more expert on dismantling the inequity (i.e., from people on the top of the coin to people on the bottom), and whose thinking and behaviour need to change in order for the inequity to be dismantled (i.e., from people on the bottom of the coin to people on the top). Reframing the problem as the ineffective and unhelpful orientation of people in positions of unearned advantage allows new possibilities for action to come to light. Below I introduce several principles as an entry point for guiding such action, i.e., for practicing critical allyship.

### Stop trying to save or fix people on the bottom of the coin

An initial step is to recognize and resist the everyday ways that people on the top of a coin unintentionally strengthen, as opposed to dismantle, the coin; that is, the things we say or do that unwittingly reflect and therefore reproduce the system of inequality. A key step in this practice is to reject the dangerous and misguided urge to save people on the oppression side of the coin, driven by the irrational sense of expertise, entitlement and access [49]. Rather, the aim of critical allyship is to operate *in solidarity with* people on the bottom of the coin. This principle applies to students, researchers or clinicians developing health promotion programs for or conducting research with marginalized communities locally or globally [50], without understanding their personal relationship to the systems of inequality that marginalize these communities in the first place [51].

This invitation to recognize the harmful effects of a fixing or saving mentality is particularly complex for those involved in clinical care, which is often defined as fixing or saving patients. The challenge is to consider how to meet individual clinical needs without reproducing systems of inequality. How might one deliver health care in a way that resists these systems and rewrites dominant narratives about power? Rehabilitation science researchers Roush and Sharby call on clinicians to consider this paradox for people with disabilities; that is, how to fix impairments while simultaneously celebrating disability as diversity [52]. Another approach is cultural safety, developed by Maori nurse, Irihapeti Ramsden, in response to the inability of the mainstream health system to meet the needs of Maori People in Aotearoa/New Zealand [53]. Cultural safety requires clinicians to understand their roles within the power differentials inherent in healthcare, including institutional discrimination, and aims to address inequities through both education and systemic change [54].

### Take active steps to learn about the systems of inequality for which one is in a position of privilege

Practicing critical allyship focuses not on the *intent* but *impact* of one’s actions, which requires deepened capacity to understand the multifaceted effects (positive and negative) of action or inaction. Thus far, I have emphasized the similarities across systems of inequality; however, the strategies by which each of these systems operate are unique. As such, a crucial step in practicing critical allyship is to actively acknowledge one’s positions of privilege and develop understanding of the logics of oppression that sustain and reproduce these inequalities; that is, the ideologies and assumptions that pervade law, policy, norms, attitudes and our everyday actions [55, 56].

A starting point for resistance is naming and discussing privilege with others on the top of that coin to diminish oblivion (i.e., to see the gorilla) and collectively build capacity for change. Examples include straight people discussing how they benefit from and reproduce heteronormativity [57], able-bodied people considering the unearned advantages they receive from ableism [58], white people learning about their role in perpetuating racism [41, 59], or settlers exploring settler identity in the context of colonialism [60, 61], with careful attention to how these structures simultaneously intersect. Each of these systems requires deep learning and, moreover, *unlearning* of entrenched assumptions to guide individual and collective action for transformative social change [62].

### Step back

Systems of inequality centre the presence, voices, needs, feelings, and worldviews of people in positions of privilege. A key step in redressing these power imbalances is for people on the top of the coin to de-centre themselves or to step back. This includes stepping back physically, such as decentring one’s privilege by literally making space for people on the oppression side of the coin at a meeting.

There is also transformative potential in stepping back metaphorically. This is the invitation to listen more and speak less. Those in positions of influence can step back to reallocate power to people who have historically been pushed to the margins. This includes making room for - and recognizing as legitimate - the feelings, approaches, and worldviews of people on the bottom of the coin. This requires those on the top of coins to demonstrate humility regarding the assumed rightness of certain ways of doing, communicating, and thinking, and stepping back to make room for alternatives.

This form of stepping back is exemplified by responses to the egregious history of Western-oriented research on Indigenous Peoples. The emergence of Indigenous research methodologies, which are consistent with an Indigenous worldview, recognize the profound violence perpetrated on Indigenous Peoples through the institutions of healthcare and research, and re-imagine research as a transformative process of cultural reclamation that is driven and controlled by Indigenous People [63–65]. This shift calls on non-Indigenous people to step back in terms of the taken-for-granted ‘truths’ embedded in a Western worldview about research and health. Following centuries of presumed entitlement and access to Indigenous bodies by non-Indigenous people, practicing critical allyship means asking oneself: What is my research to do and, importantly, what is not? [66] This reframing invites a shift from studying Indigenous people to studying whiteness, settler identity, and the roles of racism and colonialism in creating health inequities. It also calls for non-Indigenous people to only engage in research with Indigenous Peoples in the spirit of solidarity, which requires a transformative shift among non-Indigenous People who, by definition, find themselves on the top of the coin of settler colonialism [46, 67].

The goal of critical allyship is not only to change the behaviour of individuals, but to fundamentally shift the institutional arrangements that keep people up or down. To this end, stepping back involves giving up both symbolic and material power. In many cases, people on the top of the coin have the power to make material changes immediately, such as redirecting a paid speaking invitation to, or choosing to hire, a person on the bottom of the coin. Practicing allyship means looking at the material resources within one’s control (personally and professionally) and intentionally finding ways to shift those resources into the pockets of people on the bottom of the coin. This focus on redistribution of material power invites those on the top of coins to reflect critically on how far back one is prepared to step back in order to share the space and comforts accrued through unearned advantages.

### Recognize the need for action at the systemic, institutional, interpersonal and internal levels

Interpersonal interactions, in which one uses their power to intervene in moments of discrimination, are important, and often the focus of calls to “be an ally” [68]. However, practicing critical allyship requires analysis and change at multiple levels. The “critical” in critical allyship draws explicit attention to systems of power to emphasize that change at the interpersonal level is important but should not eclipse the goal of structural change.

Social structures play out through institutions like science, health care, and education, which are therefore key sites for practicing critical allyship [69, 70]. Practicing critical allyship encourages individuals to transform their own institutions [71]. For example, this approach invites reflection on how one’s academic department, professional body, or hospital may have day-to-day practices that unintentionally reflect and thereby reinforce systems of inequality. This practice requires rejecting the assumption that, for example, sexism or racism are not replicated in one’s lab, classroom or clinic, and instead proactively seeking to understand the ways that they *are,* so that the harmful effects can be mitigated [72].

Structures are deeply entrenched throughout society and embodied in its individuals. As such, a further target for analysis and change within critical allyship is the emotional, psychological and spiritual work required to deepen understanding of the intimate connections individuals hold to these systems of inequality. In other words, practicing critical allyship is more than intellectual. For people in positions of privilege, this internal work can be deeply uncomfortable but it rarely leads to lack of safety, which can be a daily threat to members of historically oppressed groups. Furthermore, this internal work is rarely role-modelled within the spheres of science and health; embracing this aspect of practicing critical allyship is therefore particularly important among influential figures within these fields.

### Do not use allyship to enhance personal power

Being in a position of privilege offers an iterative cycle of benefit: if one does nothing to dismantle the system offering unearned advantage, one continues to reap these benefits. Ironically, when one recognizes unfairness and strives to address this injustice, it is common for one’s personal or professional position to be advanced. Benefits can include approval from people on the bottom of the coin, acclaim for one’s expertise, awards for advocacy, praise for courage and selflessness (i.e., the courage to talk about issues that others have to live with daily), being hired for health equity positions, or academic promotion based on achievements in advancing health among marginalized groups.

In some cases, reward for practicing critical allyship is justified, such as publishing peer-reviewed articles or attracting grant funding based on innovative ideas or health-related interventions developed in solidarity with people on the bottom of the coin. Working in solidarity does not preclude the achievement of academic benchmarks, although there is work to be done within promotions processes to recognize the invisible, long-term and often gendered work of trust-earning that underpins equitable partnerships.

A key principle is to recognize and resist the urge to use allyship to advance one’s own power. One should not practice allyship *to be seen performing* the practice of allyship. On the contrary, one should actively resist special recognition for confronting issues that others live with every day.

This principle rejects allyship motivated by altruism, which depends on the work of oppressed groups to praise or affirm the “aspiring ally”, and which pursues justice *for* people on the bottom of the coin [26]. Rather, practicing critical allyship seeks justice for all by addressing the systems of inequality that harm everyone [73, 74]. A sign of allyship fueled by altruism (vs solidarity) is a defensive response when confronted about one’s actions or missteps [22]. Practicing critical allyship involves seeing critique as a gift, humbly admitting mistakes, and honouring the critique as a teaching to further uncover one’s own entrenched assumptions. Practicing critical allyship “sees illumination of privilege as liberating and consciously uses unearned privilege against itself” [45].

## Conclusions

The goal of this article is to help people fine-tune their capacity to see the gorilla (i.e., unearned advantage received from unfair systems of inequality) and to offer initial principles for resisting these systems. While these ideas may not yet be mainstream within the health sphere [12,75], insights regarding systems of inequality and anti-oppression are well developed in activist communities [76–78] and in other academic fields [8–11, 79, 80]. For instance, those looking to better understand sexism, heterosexism and cisgenderism can turn to gender studies [81, 82]. Those seeking to better understand ableism and ableist privilege can turn to disability studies [32, 83]. Those seeking to learn more about racism (including anti-Black racism) and white privilege can turn to the fields of critical race studies, critical ethnic studies, Black studies, and white studies [84, 85]. Scholarship on the intersection of racism and sexism is well-developed [86], including the seminal work of Crenshaw and Collins on intersectionality [37, 38]. Those working in global health can learn from the fields of postcolonialism/anti-colonialism, and feminist postcolonialism in particular, which calls into question the exclusion of non-white, non-Western perspectives within feminism [87, 88]. Furthermore, much is to be learned about the Eurocentric orientation of science and health care from the fields of Indigenous studies and Indigenous feminism [89, 90]. While these fields may not always centre health and illness as a focus of inquiry, they are crucial because these systems of inequality are such powerful determinants of health.

### Limits of and potential for the coin model

While a strength of the coin model is the simplicity of its framework for introducing complex concepts, this simplicity is also a weakness. First is the shortcoming of framing power, a complex phenomenon, in terms of simple binaries. Second, the model risks misrepresenting the intersectional and co-constituting nature of systems of inequality [44] by erroneously being construed as a simple stacking of coins. Third is the risk of assuming that all coins are the same size and have the same impact, which is not the intent of this model. For instance, racism and colonization are profoundly powerful in shaping other coins. Others have argued that classism is a system of inequality unlike all others [91]; the metaphor of the coin is helpful in reinforcing the primacy of material disparities within all systems of inequality.

A key concern is that the coin model is at risk of delinking history from these systems of inequality; that is, it does not explicitly link people in positions of privilege to their ancestors who intentionally created these systems of inequality. For instance, the coin model does not explicitly require white North Americans of Western European descent to reflect on statements such as, “I live on the traditional lands of Indigenous people that was stolen by my ancestors,” or “My wealth of today is also the result of the enslavement of Black people by my ancestors.” [92] The principles of practicing critical allyship require learning (and unlearning) about systems of inequality, including historic origins, and this important point is not foregrounded as clearly as others by this model.

A further limitation is use of the term ‘allyship’. While this term may be new to some in the health sphere, others have critiqued and moved beyond this language [93–95]. Given the audience for this model, the phrase ‘practicing critical allyship’ was deemed the least worst option, rejecting use of the noun ‘ally’, and inserting ‘critical’ to explicitly draw connection to systems of power. More important than the term are the concepts that underpin it, and the action they inform. Some will critique the principles of practicing critical allyship introduced in this article for not going far enough, and those critiques are welcome.

Finally, the coin model seeks to make visible positions of privilege, and offers principles for action for those on top of the coin. Following the tenet of stepping back, the critique rightly follows that this model centres the needs and concerns of people in positions of privilege, which stands to marginalize the oppressed. However, more than centring the needs of people in positions of privilege, this article aims to problematize the unearned advantages received by people in positions of privilege and the dire health impacts of ignoring these injustices. Further, this article is a call to action to recognize one’s positions of privilege and the imperative for reorienting one’s approach from helping unfortunate people, to working in solidarity on systems of inequality. The principles for practicing critical allyship introduced in this article offer an entry point to this reorientation; however, the practice is neither neat nor easy. Practicing allyship is fraught, messy, ongoing, and laden with missteps [46, 67, 96, 97], – but the alternative of reinforcing the status quo is far worse.

### In closing

Identifying and transcending widespread, willful oblivion regarding the harms of privilege within the health sphere is a crucial step in the path toward transformative change. As advocated by Freire over four decades ago, systems of oppression ultimately harm those on both sides of the coin (albeit in different ways) [73]. We all lose by precluding and compromising the societal contributions of talented and creative people on the bottom of the coin. Working in solidarity across the coin seeks to advance the liberation of those on both the bottom and the top. This requires a shift in orientation in line with this oft-cited quote from Indigenous elder, Lilla Watson: [98]

“If you have come here to help me, you are wasting your time. But if you have come because your liberation is bound up with mine, then let us work together.”

## Data Availability

Not applicable.
